# Molecular Analysis of Colorectal Cancers Suggests a High Frequency of Lynch Syndrome in Indonesia

**DOI:** 10.3390/cancers13246245

**Published:** 2021-12-13

**Authors:** Susanti Susanti, Satrio Wibowo, Gilang Akbariani, Naomi Yoshuantari, Didik Setyo Heriyanto, Asep Muhamad Ridwanuloh, Hariyatun Hariyatun, Adeodatus Yuda Handaya, Johan Kurnianda, Susanna Hilda Hutajulu, Mohammad Ilyas

**Affiliations:** 1 Molecular Pathology Research Group, Academic Unit of Translational Medical Science, Biodiscovery Institute, School of Medicine, University of Nottingham, Nottingham NG72UH, UK; mohammad.ilyas@nottingham.ac.uk; 2 Department of Pharmacology and Clinical Pharmacy, Faculty of Pharmacy, Universitas Muhammadiyah Purwokerto, Jawa Tengah 53182, Indonesia; 3 PathGen Diagnostik Teknologi, Center for Innovation and Utilization of Science and Technology, National Research and Innovation Agency (Badan Riset dan Inovasi Nasional/BRIN), Bogor 16911, Indonesia; satrio@pathgen.co.id (S.W.); gilang@pathgen.co.id (G.A.); 4 Department of Anatomical Pathology, Faculty of Medicine, Public Health and Nursing, Universitas Gadjah Mada, Dr. Sardjito General Hospital, Yogyakarta 55281, Indonesia; naomi.yoshuantari@mail.ugm.ac.id (N.Y.); didik_setyoheriyanto@mail.ugm.ac.id (D.S.H.); 5 Research Center for Biotechnology, National Research and Innovation Agency (BRIN), Bogor 16911, Indonesia; asep043@brin.go.id (A.M.R.); hariyatun@brin.go.id (H.H.); 6 Division of Digestive Surgeon, Department of Surgery, Faculty of Medicine, Public Health and Nursing, Universitas Gadjah Mada, Dr. Sardjito General Hospital, Yogyakarta 55281, Indonesia; yudahandaya@ugm.ac.id; 7 Division of Hematology and Medical Oncology, Department of Internal Medicine, Faculty of Medicine, Public Health and Nursing, Universitas Gadjah Mada, Dr. Sardjito General Hospital, Yogyakarta 55281, Indonesia; johan.kurnianda@ugm.ac.id (J.K.); susanna.hutajulu@ugm.ac.id (S.H.H.)

**Keywords:** microsatellite instability (MSI), BRAF mutation, MLH1 promoter methylation, high Resolution melting (HRM), early onset colorectal cancer (EOCRC)

## Abstract

**Simple Summary:**

The incidence of young people <50 years old who are diagnosed with colorectal cancer (CRC), termed as early onset colorectal cancer (EOCRC), accounted for nearly 30% of the total CRC patients in Indonesia, which is about three times higher than what is being reported in Europe, the UK and USA. Lynch syndrome (LS) is a hereditary type of CRC that is associated with a younger age of onset. Detecting LS has been long reported to be a cost-effective strategy to provide aid in the diagnosis or management of the individual or at-risk family members. The aim of this retrospective study was to screen for Lynch Syndrome in Indonesian CRC patients using simple and robust polymerase chain reaction (PCR)-based molecular testing, known as N_LyST (Nottingham Lynch Syndrome Test). To our knowledge, we are the first to study and observe a potentially higher frequency of LS (13.85%) among CRC patients in Indonesia (*n* = 231). This may partially contribute to the reported much higher rate of EOCRC found in the country.

**Abstract:**

There is about three times higher incidence of young patients <50 years old with colorectal cancer, termed EOCRC, in Indonesia as compared to Europe, the UK and USA. The aim of this study was to investigate the frequency of Lynch Syndrome (LS) in Indonesian CRC patients. The previously described Nottingham Lynch Syndrome Test (N_LyST) was used in this project. N_LyST is a robust high-resolution melting (HRM)-based test that has shown 100% concordance with standard reference methods, including capillary electrophoresis and Sanger sequencing. The test consisted of five mononucleotide microsatellite markers (BAT25, BAT26, BCAT25, MYB, EWSR1), BRAF V600E mutation and MLH1 region C promoter for methylation (using bisulphite-modified DNA). A total of 231 archival (2016–2019) formalin-fixed, paraffin-embedded (FFPE) tumour tissues from CRC patients collected from Dr. Sardjito General Hospital Yogyakarta, Indonesia, were successfully tested and analysed. Among those, 44/231 (19.05%) were MSI, 25/231 (10.82%) were harbouring BRAF V600E mutation and 6/231 (2.60%) had MLH1 promoter methylation. Almost all—186/197 (99.45%)—MSS cases were MLH1 promoter unmethylated, while there were only 5/44 (11.36%) MSI cases with MLH1 promoter methylation. Similarly, only 9/44 (20.45%) of MSI cases were BRAF mutant. There were 50/231 (21.65%) EOCRC cases, with 15/50 (30%) regarded as MSI, as opposed to 29/181 (16.02%) within the older group. In total, 32/231 patients (13.85%) were classified as “Probable Lynch” (MSI, BRAF wildtype and MLH1 promoter unmethylated), which were enriched in EOCRC as compared to older patients (24% vs. 11.05%, *p* = 0.035). Nonetheless, 30/50 (76.00%) cases among the EOCRC cases were non-LS (sporadic) and were significantly associated with a left-sided tumour. The overall survival of both “Probable Lynch” and non-LS (sporadic) groups (*n* = 227) was comparable (*p* = 0.59), with follow up period of 0–1845 days/61.5 months. Stage, node status, histological grading and ECOG score were significantly associated with patient overall survival (*p* < 0.005), yet only ECOG was an independent factor for OS (HR: 4.38; 95% CI: 1.72–11.2; *p* = 0.002). In summary, this study is the first to reveal a potentially higher frequency of LS among CRC patients in Indonesia, which may partially contribute to the reported much higher number of EOCRC as compared to the incidence in the West.

## 1. Introduction

Colorectal cancer (CRC) is one of the leading causes of cancer-related mortality. The estimated incidence of CRC worldwide in 2018 was about 1.8 million cases, comprising 11% of all cancer diagnoses, with approximately 881,000 deaths, making it the second deadliest cancer worldwide [1]. Colorectal cancer is also a major health burden in Indonesia, with an incidence of 35,000 cases per year [2]. It is the fourth most prevalent cancer, with an age-standardized annual incidence rate of 12.4/100,000 individuals and a mortality rate of 6.7/100,000 individuals [3].

In the past decade, there has been an increase of the proportion of young people (<50 years old) being diagnosed with CRC, termed as Early Onset Colorectal Cancer (EOCRC), despite the decreased incidence in the population over 50 years old [4,5,6]. This may occur in the context of inherited diseases such as Lynch Syndrome (LS), although only around 15% of EOCRC is associated with known hereditary forms of the disease [7,8,9]. Limited studies have reported that the frequency of EOCRC in Indonesia is about 30% of total CRC cases [10,11]. Our current study (under review) also confirms this interesting finding, showing approximately three times higher frequency of EOCRC in Indonesia as compared to ~10% found in western countries [12,13,14]. Nonetheless, to our knowledge, no study has been conducted to determine the frequency of Lynch Syndrome, the most common inherited type of CRC, which has been linked to a higher risk of EOCRC.

The inactivating germline mutation of the DNA MMR (mismatch repair) genes, including MLH1, MLH2, MSH2, MSH3, MSH6 or PMS2, is reported to be responsible for LS [15]. Loss of MMR results in changes in the length of microsatellites (normal segments of DNA consisting of multiple repeats of sequences 1–6 nucleotides in length, known as microsatellite instability (MSI) [16,17,18]. While germline mutations of one of the MMR genes are responsible for LS, hypermethylation silencing of MLH1 is the most common mechanism for MMR inactivation, and it is responsible for sporadic CRC tumours [19,20,21]. Thus, sporadic tumours with deficient MMR/MSI can be distinguished from tumours arising in LS by demonstrating methylation of the MLH1 promoter. Similarly, somatic mutation of BRAF is common in sporadic tumours with MSI but very rarely occurs in tumours arising in LS. BRAF V600E mutation, through inhibition of apoptosis, is an early event in pre-malignant lesions and leads to the induction of methylation in the promoter region of the MLH1 gene, resulting in the MSI phenotype and increased invasiveness of serrated pathways of sporadic CRC carcinogenesis [22,23,24].

Guidance from the National Institute of Clinical and Healthcare Excellence (NICE-UK) and National Comprehensive Cancer Network (NCCN-USA) recommends that all CRC should be screened for the possibility of LS [25,26]. The NICE pathway involves two steps: first, identify cases with deficient MMR or MSI, and then filter out sporadic cases by testing for BRAF mutation and MLH1 promoter methylation. This approach has been reported to be a cost-effective strategy, with important clinical benefits for CRC patients, particularly under 70 years old, and their relatives by implementation of appropriate surveillance pathways for early diagnosis of associated cancers. In addition, prevention measures can also be applied, such as taking a low dose of aspirin, bowel removal surgery, and lifestyle modification (smoking, weight and diet control) [27,28].

We previously introduced Nottingham Lynch Syndrome Test (N_LyST), a simple and robust screening test for LS. This single closed-tube screening panel comprises tests for microsatellite instability (MSI) and BRAF mutation MLH1 methylation promoter using polymerase chain reaction (PCR) followed by high-resolution melting (HRM) analysis [29]. N_LyST incorporates the three components of LS screening, which are testing for MSI, BRAF V600E mutation and MLH1 promoter methylation into a single PCR run. A panel of five microsatellite markers were utilised, which includes two established markers (BAT25, BAT26) and three novel markers (BCAT25, MYB and EWSR1). The MSI testing of N_LyST showed 100% concordance with immunohistochemistry (IHC) for mismatch repair protein (MMR) and capillary electrophoresis (comparable limit of detection of ~ 6.25%). The BRAF V600E mutation and MLH1 promoter methylation tests were also in agreement with Sanger sequencing [29].

In this paper, utilising the N_LyST test, we sought to screen for Lynch Syndrome among Indonesian CRC patients.

## 2. Method

### 2.1. CRC Clinical Samples

Data on 1276 consecutive cases of CRC who visited Dr. Sardjito General Hospital Yogyakarta, Indonesia, from January 2016 to December 2019 were identified from the hospital-based cancer registry. Only a total of 271 formalin-fixed, paraffin-embedded (FFPE) CRC tumour samples were available from the archives of the Anatomical Pathology Department. This was due to the hospital being the national tertiary referral hospital, where many of the patients received treatment following resection procedures that had been conducted in secondary/regional hospitals elsewhere. Patient data that were retrieved were sociodemographic (age and sex), tumour pathology (location/side, tour morphology, histological grade, TNM stage, lymphovascular status, tumour infiltrating lymphocytes/TILs) and clinical parameters (haemoglobin/Hb, albumin, body mass index/BMI and Eastern Cooperative Oncology Group/ECOG scale).

### 2.2. DNA Extraction

Genomic DNA was extracted using the QIAamp DNA FFPE tissue kit (Qiagen, USA) following the manufacturer’s protocol. DNA samples were quantified using a NanoDrop™ spectrophotometer (Thermo Scientific, Waltham, MA, USA). Samples with adequate concentration and quality were adjusted to a concentration of 20 ng/µL for PCR application.

### 2.3. Bisulphite Conversion

In order to test for methylation of the MLH1 promoter, it was necessary to modify the DNA. Bisulphite conversion of 400 ng of genomic DNA from each sample was carried out using the EZ-DNA Methylation-Lightning Kit (Zymo Research, Irvine, CA, USA), according to the manufacturer’s protocol.

### 2.4. MSI, BRAF and MLH1 Analysis Using N_LyST Panel

N_Lyst was described extensively in our previous study [29]. The method consists of detection of a panel of five mononucleotide microsatellite repeats, BRAF V600E mutations, and MLH1 region C promoter methylation status. Single-plex (single reaction for each marker) reaction was mixed with the following for a final volume of 10 µL: 1.75 µL nuclease free water (Qiagen, Germantown, MD, USA), 5 µL PCR Hot Shot Diamond PCR master mix (client Life Science, Stourbridge, UK), 1 µL Evagreen dye (Biotium, Fremont, CA, USA), 0.25 µL primers, and 2 µL DNA template. Analysis was carried out in single-step using a CFX-Connect real-time PCR instrument (Bio-Rad, Hercules CA, USA). PCR was performed under the following temperature protocol: 1 cycle of 95 °C/5 min; 45 cycles of 95 °C/10 s, 55 °C/30 s and 72 °C/30 s; and 1 cycle of 72 °C/2 min. Prior to melting analysis, PCR products underwent heating to 95 °C for 15 s, rapid cooling to 60 °C and maintenance at 60 °C for 1 min. High-resolution melting analysis was carried out by increasing temperature with an increment of 0.3 °C from 60 °C to 95 °C. The melting data were analysed following normalisation using Precision Melt Analysis (Bio-Rad, Hercules, CA, USA). For MSI analysis, samples were regarded as MSI if ≥2 markers (40%) showed instability; otherwise, they were regarded as MSS tumours. Samples showing MSI, BRAF mutation and MLH1 promoter methylation were classified as “Probable Lynch”.

### 2.5. Statistical Analysis

Correlation between variables was calculated using the Fisher’s exact test with a significance level of α = 0.05. The Kaplan-Meier method was used to calculate the overall survival (OS), and comparisons between groups of interest were carried out using a log-rank test. A multivariate analysis of the factors that influence the OS was performed using the Cox proportional hazards regression model. All analysis was performed using R version 4.0.3.

## 3. Results

### 3.1. Patient Clinicopathology Characteristics

As shown in Table 1, there were mostly adenocarcinoma (97.84%) among the 231 CRC samples with complete molecular data, with an equal number of female and male (51.52% vs. 48.48%), enrichment of EOCRC (21.65%), higher frequency of left-sided tumours that included the rectum (77.92%), advanced stages of III (24.24%) and IV (39.83%) as well as T3 (64.94%) and T4 (22.94%). Yet, the majority of the samples had a lower histology grade of 1 and 2 (44.59% and 39.39%, respectively), with almost equal distribution between with and without nodal involvement and metastasis. Most cases showed an Hb level of ≥10 g/dL (84.85%) and ECOG scale of 0–1 (63.64%).

### 3.2. Lynch Syndrome Screening Using N_Lyst

**Figure 1:**
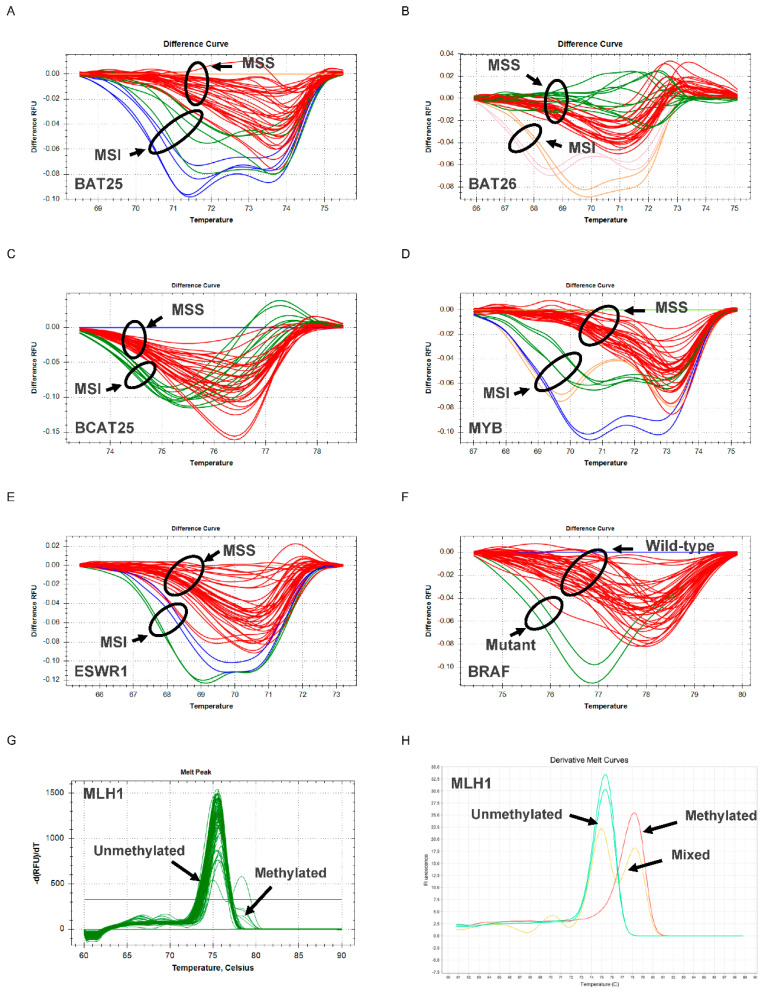
Use of the N_LyST panel to screen for Lynch syndrome. Differential plots are shown for 5 microsatellite markers BAT25 (**A**), BAT26 (**B**), BCAT25 (**C**), MYB (**D**), and EWSR1 (**E**). The differential melt curves of the tumours with microsatellite instability (MSI) and those that are microsatellite stable (MSS) are indicated by the distinct black circles. The differential melt curves of the tumours with wild-type BRAF are different from those with mutant BRAF, as indicated by respective black circles (**F**). Derivative plots are shown for the CpG Island region C of MLH1 for tumour samples (**G**) and human DNA control (**H**). For tumour samples, two discrete melting forms are shown: ‘methylated’, comprising two melting peaks that represent methylated DNA (from tumour epithelium) and non-methylated DNA (from tumour stroma) or ‘non-methylated’, comprising one melting peak that characterises a completely non-methylated tumour and stroma cell population.

MSI, BRAF and MLH1 promoter methylation analysis was successfully done on 231 samples. The remaining 40 samples were not included in the PCR testing due to low quality of DNA following FFPE tissue extraction and bisulphite conversion. The typical HRM plots for the N_LyST panel are shown in Figure 1.

MSI status was determined based on a threshold of two unstable MSI markers. Among the 231 samples, the majority of the MSI samples, 40/44 (90.91%), showed instability on four to five markers. Likewise, most of the MSS samples, 166/181 (88.77%), showed no instability in any of the MSI markers (Table 2).

Though there was a significant correlation between MSI and BRAF mutation (*p* = 0.031), only 9/44 (20.45%) of MSI cases were BRAF mutant. The mutation frequency was even less in MSS, accounting for 16/187 (8.56%). Almost all, 186/197 (99.45%), MSS cases were MLH1 promoter unmethylated, while 5/44 (11.36%) of MSI cases had MLH1 promoter methylation (*p* = 0.001) (Table 3).

The results summarised in Table 4 show that among 231 samples with complete molecular information, 44/231 (19.05%) were MSI, 25/231 (10.82%) were harbouring BRAF V600E mutation and 6/231 (2.60%) had MLH1 promoter methylation. Based on the workflow recommended by NICE, samples that have MSI, wild-type BRAF and unmethylated MLH1 promoter based on the N_LyST test were classified “Probable Lynch”. Only 3/35 (8.6%) of MSI cases with no mutation of BRAF V600E showed MLH1 promoter methylation. Thus, there were 32 samples (13.85%) identified as “Probable Lynch”. The frequency of MSI and “Probable Lynch” status among those of EOCRC (under 50 years old) were significantly higher compared to the older patients (30% vs. 16.02%, *p* = 0.040 and 24% vs. 11.05%, *p* = 0.035, respectively). Nonetheless, there were 38/50 (76.00%) cases among the EOCRC that were considered as non-LS (sporadic) CRC.

### 3.3. Clinicopathology-Molecular Characteristic Association and Survival

In general, as shown in Table 5, there was no significant association between many patients’ clinicopathology characteristics and the molecular features (*n* = 231). Nevertheless, this study found a significant association of right-sided tumour with MSI (*p* < 0.001), methylated MLH1 promoter (*p* = 0.047), and less frequent “Probable Lynch” status (*p* = 0.003). Higher histology grading (3 and 4) was associated with MSI (*p* = 0.003) and MLH1 promoter methylation (*p* = 0.004). Although observed in only small number of samples, mucinous tumours were positively associated with MSI (*p* = 0.049) and lower frequency of “Probable Lynch” status (*p* = 0.020). Similarly, a low level of Hb (<10 g/dL) was also associated with MSI (*p* = 0.044) and lower frequency of “Probable Lynch” status (*p* = 0.010). On the contrary, higher ECOG was associated with MSI and higher frequency of “Probable Lynch” status (*p* = 0.010).

**Figure 2:**
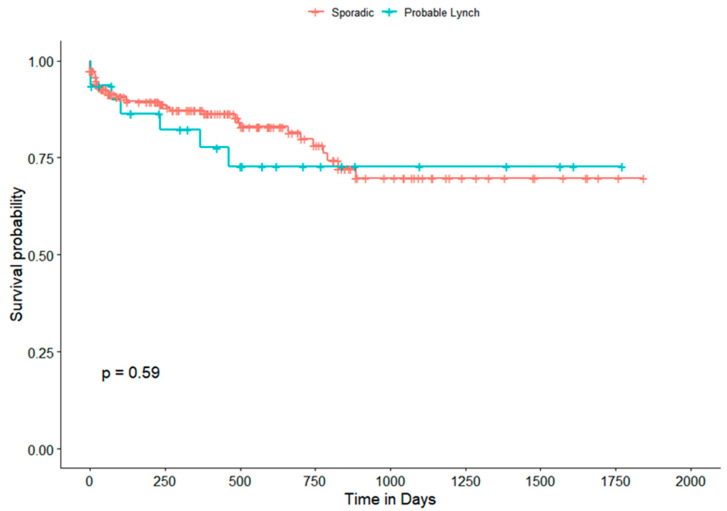
Overall survival curves for CRC patients in “Probable Lynch” and sporadic groups. Survival was evaluated using Kaplan–Meier curves and compared with the log-rank test.

The overall survival (follow-up period of 0–1845 days/61.5 months) of both “Probable Lynch” and non-LS (sporadic) groups (*n* = 227) was comparable (*p* = 0.59) (Figure 2). In univariate analysis, there were significant associations between stage (*p* = 0.040), node status (*p* = 0.005), histological grading (*p* = 0.036) and ECOG (*p* ≤ 0.001) with patient overall survival. Multivariate analysis using the Cox proportional hazards model further showed ECOG (*p* = 0.002) as an independent survival predictor in the CRC population (Table 6).

## 4. Discussion

The molecular cohort tested in this study was enriched for 21.65% of EOCRC (<50 years old). This was part of a larger study to assess the clinicopathology parameters of 1276 consecutive patients treated for CRC at Dr Sardjito Hospital, Yogyakarta, Indonesia, during 2016–2019. Despite only limited FFPE samples being available for molecular testing in this project, the patient clinicopathology characteristics of this sub-population for molecular testing were similar to the characteristics found in the overall CRC cohort (Supplementary Table S1). The frequency of EOCRC in the larger cohort was 27.6% [30], as similarly reflected in the molecular cohort. This high proportion of EOCRC was intriguing, as no existing data have been reported regarding the extent of LS, which is usually characterised by early onset, in the local context of Indonesia.

In this study, MSI and BRAF frequency (19.05% and 10.82%) among the CRC cases was within the range of previously reported data, at about ~20% and ~15%, respectively [31,32,33,34,35,36,37]. The well-established strong association (*p* = 0.031) between MSI and BRAF V600E mutation was also confirmed in this study, although the percentage (20.45%) was much lower than what usually has been reported: 50–70% [29,38,39]. Yet, the frequency of BRAF mutation in MSS cases (8.56%) was similar to that reported in the literature [24,40]. Lower BRAF mutation frequency in CRC in Asian countries such as Japan, Russia, Israel and China has been reported previously by many, ranging from 3–6% of all cases and about 15.4% of cases with loss of the expression of MLH1/PMS2 [33,41,42,43,44,45,46,47].

There was also a much lower frequency of MLH1 promoter methylation, at only 2.60%, compared to approximately 20% reported in the published literature [48,49]. Colorectal cancer showing somatic MLH1 methylation tends to occur at relatively advanced ages, is more common in women, is often located in the right colon (as also shown in this study), displays the somatic activating BRAF V600E mutation and occurs in sporadic tumours instead of in the inherited form of CRC, like LS [24,50,51]. However, it has been reported on very rare occasion that MLH1 promoter methylation could present as a constitutional defect, detectable in normal cells such as peripheral leukocytes, in patients presenting with phenotypic characteristics of LS [52].

This study reveals much higher “Probable Lynch” (13.85%) as compared to what has been reported in western countries, where LS accounts for about 2–3% of total CRC cases [53,54,55]. This was indicated by the presence of MSI with a much lower number of BRAF V600E mutations and MLH1 promoter methylations. It would be ideal to confirm the findings by analysing the germline MMR mutation. Nonetheless, we are confident of the robustness of the N_LyST tests as detailed in the previous publication, describing the high frequency of BRAF mutants and MLH1 methylation within the MSI of the UK CRC samples, with near perfect agreement with some standard methods [29]. Prior validation of the test using 50 Indonesian CRC samples with known MSI, BRAF and MLH1 promoter methylation status also showed 100% concordance (unpublished data). In this study, the fact that ~90% of MSI samples showed instability in more than four MSI markers and ~90% of MSS samples showed no instability in any of the markers implies that it was less likely that the instabilities observed were due to polymorphism. Further, BRAF V600E mutation, and MLH1 promoter ‘C region’ methylation specifically (as in the N_LyST panel), have been reported as strong predictors of negative MMR mutation status [56].

As expected, the “Probable Lynch” was more frequent in EOCRC as compared to the older group, accounting for 24% of all EOCRC, which is slightly higher than the 16.1% that was reported in a study conducted on Asian immigrants with CRC in the USA [57]. Yet, the majority of EOCRC was non-LS, referred as sporadic cases. Despite the global trend of increased incidence, studies comparing EOCRC and LOCRC show no difference in the profile or frequency of somatic mutation in the known cancer driver genes [9,58,59]. This study found that non-LS cases were associated with left-sided tumours, which has also been reported by others [60,61,62]. The sporadic cases also were associated with low levels of Hb, which has been suggested to be closely associated with a higher stage of disease at presentation and a higher risk of death [63].

Despite previously reports in the literature that LS patients have better long-term survival prognosis than sporadic CRC patients [64,65,66], comparable overall survival was observed in this study between the “Probable Lynch” and the non-LS (sporadic) groups. Consistent with the findings from the larger cohort of CRC (*n* = 1276) (Hutajulu et al., under review) and with previous reports on selected cases of CRC of local patients, ECOG index played an important role in predicting the OS [67].

Detecting LS has been long reported to provide aid in the diagnosis or management of the individual or at-risk family members, including the potential of non-invasive CRC prevention with aspirin [68]. It is also worth noting that there was a considerable number of “Probable Lynch” cases among the older patients (11.05%), which supports the cost-effectiveness of LS screening for all CRC patients up to 70 years old [27,28]. MSI testing is likely to increase, as it provides information that extends beyond LS testing; for example, MSI can be used to stratify patients into groups eligible for treatment with 5-fluorouracil-based therapy [69,70], which is the most frequent chemotherapy regimen for CRC in Indonesia, or the latest immunotherapy such as pembrolizumab [71].

We found only 3/35 (8.6%) cases of MSI with BRAF wildtype group showed MLH1 promoter methylation, which are less likely to be LS. This is in contrast to a recent study in China, where 37% of cases without BRAF V600E mutation had MLH1 promoter methylation [47]. This suggests, at least in Indonesia, the prospective of simplifying the diagnostic test workflow to MSI and BRAF testing only, without the need for performing the bisulphite conversion for MLH1 promoter methylation analysis, preferably with the inclusion of family history of CRC as additional criteria. This study showed that N_LyST has high utility to accommodate low-quality samples and portability, as it does not require expensive machines and has minimal infrastructure requirements, which fits the need for implementation in recourse limited settings. Obviously, further validation using larger cohorts and confirmation of the germline mutation of the relevant MMR genes are required.

## 5. Conclusions

In summary, despite some limitations, which include relatively a small number of samples from a single centre and the absence of germline mutation data on the MMR genes, to our knowledge, this study is the first to reveal a potentially higher incidence of Lynch Syndrome among CRC patients in Indonesia, which may partially contribute to the reported much higher number of EOCRC found in some studies. Further study is required to examine the extent of LS incidence in Indonesia, including in the population setting, to confirm the existing and novel germline pathological variants. Furthermore, a large proportion of EOCRC, as shown in this study, was considered sporadic (non-LS), for which the mechanism of the so-called “accelerated carcinogenesis” warrants further investigation.

## Figures and Tables

**Table 1:** Patient clinicopathology characteristics.

Characteristic	*n* = 231
Age	
<50	50 (21.65%)
≥50	181 (78.35%)
Sex	
Female	119 (51.52%)
Male	112 (48.48%)
Tumor Site	
Left	180 (77.92%)
Right	50 (21.65%)
Unknown	1 (0.43%)
Stage	
I	11 (4.76%)
II	66 (28.57%)
III	56 (24.24%)
IV	92 (39.83%)
Unknown	6 (2.60%)
T Status	
1	2 (0.87%)
2	25 (10.82%)
3	150 (64.94%)
4	53 (22.94%)
x	1 (0.43%)
N Status	
0	115 (49.78%)
1	80 (34.63%)
2	30 (12.99%)
x	6 (2.60%)
Metastatic Status	
0	134 (58.01%)
1	91 (39.39%)
x	6 (2.60%)
Histological Grading	
1	103 (44.59%)
2	91 (39.39%)
3	32 (13.85%)
4	2 (0.87%)
Unknown	3 (1.30%)
Lymphovascular Status	
0	51 (22.08%)
1	58 (25.11%)
Unknown	122 (52.81%)
Pathological Morphology	
Adenocarcinoma	226 (97.84%)
Mucinous Carcinoma	5 (2.16%)
TILs	
Low	42 (18.18%)
Medium	74 (32.03%)
High	76 (32.90%)
Unknown	39 (16.88%)
Hemoglobin level (g/dL)	
<10	27 (11.69%)
≥10	196 (84.85%)
Unknown	8 (3.46%)
Serum albumin (g/dL)	
<3.5	98 (42.42%)
>3.5	64 (27.71%)
Unknown	69 (29.87%)
ECOG	
ECOG 0–1	147 (63.64%)
ECOG 2	36 (15.58%)
ECOG 3–4	19 (8.23%)
Unknown	29 (12.55%)
BMI (kg/m^2^)	
<18.5	71 (30.74%)
18.5–22.9	90 (38.96%)
23–24.9	31 (13.42%)
≥25	30 (12.99%)
Unknown	9 (3.90%)

**Table 2:** Microsatellite marker instability distribution (*N* = 231).

MSI Status	Number of Unstable MSI Markers					
	0	1	2	3	4	5
MSI	(0%)	(0%)	3 (6.82%)	1 (2.27%)	7 (15.91%)	33 (75%)
MSS	166 (88.77%)	21 (11.23%)	(0%)	(0%)	(0%)	(0%)

MSI, Microsatellite Instability; MSS, Microsatellite Stable.

**Table 3:** Association between MSI, BRAF mutation and MLH1 promoter methylation (*n* = 231).

Characteristic	Microsatellite Instability		
	MSI, *N* = 44	MSS, *N* = 187	*p*-Value ^1^
BRAF Exon 15			0.031 *
Mutant	9 (20.45%)	16 (8.56%)	
Wild-type	35 (79.55%)	171 (91.44%)	
MLH1 Methylation			0.001 **
Methylated	5 (11.36%)	1 (0.53%)	
Unmethylated	39 (88.64%)	186 (99.47%)	
	BRAF Exon 15		
	Mutant, *N* = 25	Wild-type, *N* = 206	*p*-Value
MLH1 Methylation			0.13
Methylated	2 (8.00%)	4 (1.94%)	
Unmethylated	23 (92.00%)	202 (98.06%)	

^1^ Fisher’s exact test; * *p* < 0.05; ** *p* < 0.01.

**Table 4:** Molecular features by N_LyST panel.

Characteristic	Total	Age Group		
	*N* = 231	<50, *N* = 50 ^1^	≥50, *N* = 181	*p*-Value ^1^
Microsatellite Instability Status				0.040 *
MSI	44 (19.05%)	15 (30.00%)	29 (16.02%)	
MSS	187 (80.95%)	35 (70.00%)	152 (83.98%)	
BRAF Exon 15				0.6
Mutant	25 (10.82%)	4 (8.00%)	21 (11.60%)	
Wild-type	206 (89.18%)	46 (92.00%)	160 (88.40%)	
MLH1 Methylation				>0.9
Methylated	6 (2.60%)	1 (2.00%)	5 (2.76%)	
Unmethylated	225 (97.40%)	49 (98.00%)	176 (97.24%)	
Probable Lynch				0.035 *
No	199 (86.15%)	38 (76.00%)	161 (88.95%)	
Yes	32 (13.85%)	12 (24.00%)	20 (11.05%)	

^1^ Fisher’s exact test; * *p* < 0.05; MSI, Microsatellite Instability; MSS, Microsatellite Stable.

**Table 5:** Association between clinicopathology characteristics and molecular features (*n* = 231).

Characteristic	Microsatellite Instability Status			BRAF Exon 15			MLH1 Promoter			Probable Lynch		
	MSI *N* = 44	MSS *N* = 187	*p*-Value ^1^	Mutant *N* = 25	Wild-Type *N* = 206	*p*-Value ^1^	Methylated *N* = 6	Unmethylated *N* = 225	*p*-Value ^1^	No *N* = 199	Yes *N* = 32	*p*-Value ^1^
Sex			0.4			0.7			0.7			0.13
Female	20 (45.45%)	99 (52.94%)		14 (56.00%)	105 (50.97%)		4 (66.67%)	115 (51.11%)		107 (53.77%)	12 (37.50%)	
Male	24 (54.55%)	88 (47.06%)		11 (44.00%)	101 (49.03%)		2 (33.33%)	110 (48.89%)		92 (46.23%)	20 (62.50%)	
Tumor Site			<0.001 ***			0.14			0.047 *			0.003 **
Left	23 (52.27%)	157 (83.96%)		19 (76.00%)	161 (78.16%)		2 (33.33%)	178 (79.11%)		162 (81.41%)	18 (56.25%)	
Right	20 (45.45%)	30 (16.04%)		5 (20.00%)	45 (21.84%)		4 (66.67%)	46 (20.44%)		36 (18.09%)	14 (43.75%)	
Unknown	1 (2.27%)	0 (0.00%)		1 (4.00%)	0 (0.00%)		0 (0.00%)	1 (0.44%)		1 (0.50%)	0 (0.00%)	
Stage			0.4			0.7			0.7			0.6
I	3 (6.82%)	8 (4.28%)		0 (0.00%)	11 (5.34%)		0 (0.00%)	11 (4.89%)		8 (4.02%)	3 (9.38%)	
II	10 (22.73%)	56 (29.95%)		6 (24.00%)	60 (29.13%)		1 (16.67%)	65 (28.89%)		58 (29.15%)	8 (25.00%)	
III	14 (31.82%)	42 (22.46%)		6 (24.00%)	50 (24.27%)		3 (50.00%)	53 (23.56%)		48 (24.12%)	8 (25.00%)	
IV	15 (34.09%)	77 (41.18%)		12 (48.00%)	80 (38.83%)		2 (33.33%)	90 (40.00%)		80 (40.20%)	12 (37.50%)	
Unknown	2 (4.55%)	4 (2.14%)		1 (4.00%)	5 (2.43%)		0 (0.00%)	6 (2.67%)		5 (2.51%)	1 (3.12%)	
T Status			0.7			>0.9			0.4			0.6
1	1 (2.27%)	1 (0.53%)		0 (0.00%)	2 (0.97%)		0 (0.00%)	2 (0.89%)		1 (0.50%)	1 (3.12%)	
2	4 (9.09%)	21 (11.23%)		3 (12.00%)	22 (10.68%)		0 (0.00%)	25 (11.11%)		22 (11.06%)	3 (9.38%)	
3	30 (68.18%)	120 (64.17%)		16 (64.00%)	134 (65.05%)		6 (100.00%)	144 (64.00%)		130 (65.33%)	20 (62.50%)	
4	9 (20.45%)	44 (23.53%)		6 (24.00%)	47 (22.82%)		0 (0.00%)	53 (23.56%)		45 (22.61%)	8 (25.00%)	
x	0 (0.00%)	1 (0.53%)		0 (0.00%)	1 (0.49%)		0 (0.00%)	1 (0.44%)		1 (0.50%)	0 (0.00%)	
N Status			0.8			0.7			0.3			0.8
0	21 (47.73%)	94 (50.27%)		11 (44.00%)	104 (50.49%)		1 (16.67%)	114 (50.67%)		97 (48.74%)	18 (56.25%)	
1	15 (34.09%)	65 (34.76%)		10 (40.00%)	70 (33.98%)		4 (66.67%)	76 (33.78%)		70 (35.18%)	10 (31.25%)	
2	6 (13.64%)	24 (12.83%)		3 (12.00%)	27 (13.11%)		1 (16.67%)	29 (12.89%)		27 (13.57%)	3 (9.38%)	
x	2 (4.55%)	4 (2.14%)		1 (4.00%)	5 (2.43%)		0 (0.00%)	6 (2.67%)		5 (2.51%)	1 (3.12%)	
Metastatic Status			0.4			0.4			>0.9			>0.9
0	27 (61.36%)	107 (57.22%)		12 (48.00%)	122 (59.22%)		4 (66.67%)	130 (57.78%)		115 (57.79%)	19 (59.38%)	
1	15 (34.09%)	76 (40.64%)		12 (48.00%)	79 (38.35%)		2 (33.33%)	89 (39.56%)		79 (39.70%)	12 (37.50%)	
x	2 (4.55%)	4 (2.14%)		1 (4.00%)	5 (2.43%)		0 (0.00%)	6 (2.67%)		5 (2.51%)	1 (3.12%)	
Histological Grading			0.003 **			0.8			0.004 **			0.14
1	12 (27.27%)	91 (48.66%)		10 (40.00%)	93 (45.15%)		2 (33.33%)	101 (44.89%)		94 (47.24%)	9 (28.12%)	
2	18 (40.91%)	73 (39.04%)		10 (40.00%)	81 (39.32%)		0 (0.00%)	91 (40.44%)		76 (38.19%)	15 (46.88%)	
3	14 (31.82%)	18 (9.63%)		5 (20.00%)	27 (13.11%)		3 (50.00%)	29 (12.89%)		24 (12.06%)	8 (25.00%)	
4	0 (0.00%)	2 (1.07%)		0 (0.00%)	2 (0.97%)		1 (16.67%)	1 (0.44%)		2 (1.01%)	0 (0.00%)	
Unknown	0 (0.00%)	3 (1.60%)		0 (0.00%)	3 (1.46%)		0 (0.00%)	3 (1.33%)		3 (1.51%)	0 (0.00%)	
Lymphovascular Status			0.3			0.8			0.7			0.3
0	11 (25.00%)	40 (21.39%)		4 (16.00%)	47 (22.82%)		2 (33.33%)	49 (21.78%)		42 (21.11%)	9 (28.12%)	
1	14 (31.82%)	44 (23.53%)		6 (24.00%)	52 (25.24%)		1 (16.67%)	57 (25.33%)		48 (24.12%)	10 (31.25%)	
Unknown	19 (43.18%)	103 (55.08%)		15 (60.00%)	107 (51.94%)		3 (50.00%)	119 (52.89%)		109 (54.77%)	13 (40.62%)	
Pathological Morphology			0.049 *			>0.9			>0.9			0.020 *
Adenocarcinoma	41 (93.18%)	185 (98.93%)		25 (100.00%)	201 (97.57%)		6 (100.00%)	220 (97.78%)		197 (98.99%)	29 (90.62%)	
Mucinous Carcinoma	3 (6.82%)	2 (1.07%)		0 (0.00%)	5 (2.43%)		0 (0.00%)	5 (2.22%)		2 (1.01%)	3 (9.38%)	
TILs			0.4			0.7			0.14			0.12
Low	10 (22.73%)	32 (17.11%)		3 (12.00%)	39 (18.93%)		2 (33.33%)	40 (17.78%)		34 (17.09%)	8 (25.00%)	
Medium	15 (34.09%)	59 (31.55%)		7 (28.00%)	67 (32.52%)		0 (0.00%)	74 (32.89%)		61 (30.65%)	13 (40.62%)	
High	10 (22.73%)	66 (35.29%)		11 (44.00%)	65 (31.55%)		2 (33.33%)	74 (32.89%)		71 (35.68%)	5 (15.62%)	
Unknown	9 (20.45%)	30 (16.04%)		4 (16.00%)	35 (16.99%)		2 (33.33%)	37 (16.44%)		33 (16.58%)	6 (18.75%)	
Hemoglobin level (g/dL)			0.044 *			0.8			0.12			0.010 **
<10	10 (22.73%)	17 (9.09%)		2 (8.00%)	25 (12.14%)		1 (16.67%)	26 (11.56%)		18 (9.05%)	9 (28.12%)	
≥10	33 (75.00%)	163 (87.17%)		22 (88.00%)	174 (84.47%)		4 (66.67%)	192 (85.33%)		173 (86.93%)	23 (71.88%)	
Unknown	1 (2.27%)	7 (3.74%)		1 (4.00%)	7 (3.40%)		1 (16.67%)	7 (3.11%)		8 (4.02%)	0 (0.00%)	
Serum albumin (g/dL)			0.4			0.9			0.3			0.9
<3.5	16 (36.36%)	82 (43.85%)		10 (40.00%)	88 (42.72%)		3 (50.00%)	95 (42.22%)		85 (42.71%)	13 (40.62%)	
>3.5	11 (25.00%)	53 (28.34%)		8 (32.00%)	56 (27.18%)		0 (0.00%)	64 (28.44%)		56 (28.14%)	8 (25.00%)	
Unknown	17 (38.64%)	52 (27.81%)		7 (28.00%)	62 (30.10%)		3 (50.00%)	66 (29.33%)		58 (29.15%)	11 (34.38%)	
ECOG			0.043 *			0.2			0.5			0.010 **
ECOG 0–1	24 (54.55%)	123 (65.78%)		18 (72.00%)	129 (62.62%)		4 (66.67%)	143 (63.56%)		130 (65.33%)	17 (53.12%)	
ECOG 2	12 (27.27%)	24 (12.83%)		1 (4.00%)	35 (16.99%)		0 (0.00%)	36 (16.00%)		25 (12.56%)	11 (34.38%)	
ECOG 3–4	1 (2.27%)	18 (9.63%)		1 (4.00%)	18 (8.74%)		1 (16.67%)	18 (8.00%)		19 (9.55%)	0 (0.00%)	
Unknown	7 (15.91%)	22 (11.76%)		5 (20.00%)	24 (11.65%)		1 (16.67%)	28 (12.44%)		25 (12.56%)	4 (12.50%)	
BMI (kg/m^2^)			0.2			0.4			0.4			0.2
<18.5	19 (43.18%)	52 (27.81%)		6 (24.00%)	65 (31.55%)		4 (66.67%)	67 (29.78%)		57 (28.64%)	14 (43.75%)	
18.5–22.9	16 (36.36%)	74 (39.57%)		14 (56.00%)	76 (36.89%)		1 (16.67%)	89 (39.56%)		79 (39.70%)	11 (34.38%)	
23–24.9	6 (13.64%)	25 (13.37%)		3 (12.00%)	28 (13.59%)		0 (0.00%)	31 (13.78%)		26 (13.07%)	5 (15.62%)	
≥25	2 (4.55%)	28 (14.97%)		1 (4.00%)	29 (14.08%)		1 (16.67%)	29 (12.89%)		29 (14.57%)	1 (3.12%)	
Unknown	1 (2.27%)	8 (4.28%)		1 (4.00%)	8 (3.88%)		0 (0.00%)	9 (4.00%)		8 (4.02%)	1 (3.12%)	

^1^ Fisher’s exact test; * *p* < 0.05; ** *p* < 0.01; *** *p* < 0.001.

**Table 6:** Univariate and multivariate survival analysis (*n* = 231).

Characteristic		Univariate				Multivariate			
	N	Event N	HR ^1^	95% CI ^1^	*p*-Value	Event N	HR ^1^	95% CI ^1^	*p*-Value
Age	227	40							
<50			—	—					
≥50			0.85	0.42, 1.74	0.7				
Sex	227	40							
Female			—	—					
Male			0.90	0.48, 1.69	0.7				
Tumor Site	226	40							
Left			—	—					
Right			1.01	0.48, 2.12	>0.9				
Stage	223	40				30			
I–II			—	—			—	—	
III–IV			2.18	1.03, 4.60	0.040 *		1.82	0.58, 5.73	0.3
T Status	227	40							
1–2			—	—					
3–4			3.21	0.77, 13.3	0.11				
x			^†^						
Node Status	227	40				30			
0			—	—			—	—	
1			1.29	0.64, 2.61	0.5		0.79	0.27, 2.37	0.7
2			3.35	1.45, 7.72	0.005 **		1.97	0.62, 6.30	0.3
x			^†^						
Metastatic Status	227	40							
0			—	—					
1			1.86	1.00, 3.48	0.051				
x			^†^						
Histological Grading	225	38				30			
1–2			—	—			—	—	
3–4			2.23	1.05, 4.71	0.036 *		1.27	0.51, 3.16	0.6
Lymphovascular Status	109	22							
0			—	—					
1			0.70	0.30, 1.65	0.4				
Pathological Morphology	227	40							
Adenocarcinoma			—	—					
Mucinous Carcinoma			1.66	0.23, 12.1	0.6				
TILs	189	33							
High			—	—					
Medium			0.85	0.38, 1.93	0.7				
Low			2.29	0.95, 5.51	0.065				
Hemoglobin level (g/dL)	220	40							
<10			—	—					
≥10			0.62	0.22, 1.79	0.4				
Serum albumin (g/dL)	160	35							
<3.5			—	—					
>3.5			0.62	0.31, 1.25	0.2				
ECOG	198	32				30			
ECOG 0–1			—	—			—	—	
ECOG 2			2.18	0.90, 5.27	0.083		1.70	0.64, 4.50	0.3
ECOG 3–4			4.60	1.98, 10.7	<0.001 ***		4.38	1.72, 11.2	0.002 **
BMI (kg/m^2^)	219	38							
<18.5			—	—					
18.5–22.9			0.87	0.41, 1.84	0.7				
23–24.9			0.81	0.28, 2.29	0.7				
≥25			0.73	0.26, 2.07	0.6				
Microsatellite Instability Status	227	40							
MSI			—	—					
MSS			0.82	0.39, 1.73	0.6				
BRAF Exon 15	227	40							
Mutant			—	—					
Wild-type			0.89	0.35, 2.28	0.8				
MLH1 Methylation	227	40							
Methylated			—	—					
Unmethylated			0.96	0.13, 6.96	>0.9				
Probable Lynch	227	40							
No			—	—					
Yes			1.25	0.55, 2.83	0.6				

^1^ HR = Hazard Ratio, CI = Confidence Interval; ^†^ Too small a number to analyse; TILs, Tumour-infiltrating lymphocytes; ECOG, Eastern Cooperative Oncology Group Performance Status; BMI, Body Mass Index; MSI, Microsatellite Instability; MSS, Microsatellite Stable; * *p* < 0.05; ** *p* < 0.01; *** *p* < 0.001.

## Supplementary Materials

The following are available online at https://www.mdpi.com/article/10.3390/cancers13246245/s1, Table S1: Clinicopathology characteristics of overall Jogjakarta CRC cohort and the sub-population used for molecular testing.
