# Supplementary material for: Molecular Analysis of Colorectal Cancers Suggests a High Frequency of Lynch Syndrome in Indonesia
Source: Cancers (Basel). 2021 Dec 13;13(24):6245. doi: 10.3390/cancers13246245 (PMC8699188; doi:10.3390/cancers13246245)
Supplement: Supplementary file 1 [file cancers-13-06245-s001.zip › cancers-1487965-supplementary.pdf]

Table S1. Clinicopathology characteristics of overall Jogjakarta CRC cohort and the sub-population used for molecular testing.

Characteristic	Population, N = 1,276	Subpopulation, N = 231
Age		
<50	353 (27.64%)	50 (21.65%)
>=50	924 (72.36%)	181 (78.35%)
Sex		
Female	589 (46.12%)	119 (51.52%)
Male	688 (53.88%)	112 (48.48%)
Tumor Site		
Left	948 (74.24%)	180 (77.92%)
Right	268 (20.99%)	50 (21.65%)
Unknown	61 (4.78%)	1 (0.43%)
Stage		
I	29 (2.27%)	11 (4.76%)
II	177 (13.86%)	66 (28.57%)
III	127 (9.95%)	56 (24.24%)
IV	441 (34.53%)	92 (39.83%)
Unknown	503 (39.39%)	6 (2.60%)
T Status		
1	21 (1.64%)	2 (0.87%)
2	111 (8.69%)	25 (10.82%)
3	645 (50.51%)	150 (64.94%)
4	231 (18.09%)	53 (22.94%)
x	269 (21.06%)	1 (0.43%)
N Status		
0	412 (32.26%)	115 (49.78%)
1	287 (22.47%)	80 (34.63%)
2	95 (7.44%)	30 (12.99%)
x	483 (37.82%)	6 (2.60%)
Metastatic Status		
0	352 (27.56%)	134 (58.01%)
1	440 (34.46%)	91 (39.39%)
x	485 (37.98%)	6 (2.60%)
Histological Grading		
1	511 (40.02%)	103 (44.59%)
2	343 (26.86%)	91 (39.39%)
3	119 (9.32%)	32 (13.85%)
4	5 (0.39%)	2 (0.87%)
Unknown	299 (23.41%)	3 (1.30%)
Pathological Morphology		
Adenocarcinoma	1,115 (87.31%)	226 (97.84%)
Mucinous Carcinoma	53 (4.15%)	5 (2.16%)
Signet Ring Cell Carcinoma	25 (1.96%)	0 (0.00%)
Other	84 (6.58%)	0 (0.00%)
Hemoglobin level (g/dL)		
<10	253 (19.81%)	27 (11.69%)
>=10	857 (67.11%)	196 (84.85%)
Unknown	167 (13.08%)	8 (3.46%)
Serum albumin (g/dL)		
<3,5	398 (31.17%)	98 (42.42%)
>3,5	302 (23.65%)	64 (27.71%)
Unknown	577 (45.18%)	69 (29.87%)
ECOG		
ECOG 0-1	717 (56.15%)	147 (63.64%)
ECOG 2	203 (15.90%)	36 (15.58%)
ECOG 3-4	116 (9.08%)	19 (8.23%)
Unknown	241 (18.87%)	29 (12.55%)
BMI (kg/m^2)		
<18,5	364 (28.50%)	71 (30.74%)
18,5-22,9	529 (41.43%)	90 (38.96%)
23-24,9	132 (10.34%)	31 (13.42%)
>=25	116 (9.08%)	30 (12.99%)
Unknown	136 (10.65%)	9 (3.90%)

ECOG, Eastern Cooperative Oncology Group Performance Status; BMI, Body Mass Index
